# Biased by our imaginings

**DOI:** 10.1111/mila.12225

**Published:** 2018-11-28

**Authors:** Ema Sullivan‐Bissett

**Affiliations:** ^1^ Department of Philosophy University of Birmingham Birmingham UK

**Keywords:** association, belief, folk psychology, imagination, implicit bias, unconscious imagination

## Abstract

I propose a new model of implicit bias, according to which implicit biases are constituted by unconscious imaginings. I argue that my model accommodates characteristic features of implicit bias, does not face the problems of the doxastic model, and is uniquely placed to accommodate the structural heterogeneity in the category of implicit bias. Finally I turn to how my view relates to holding people accountable for their biases and what we know about intervention strategies.

## 1 INTRODUCTION

In this paper I argue that implicit biases are constituted by unconscious imaginings. I begin by endorsing a principle of parsimony when confronted with unfamiliar phenomena. I introduce implicit bias in terms congenial to what most philosophers and psychologists have said about their nature in the literature so far, before moving to a discussion of the doxastic model of implicit bias and objections to it. I then introduce unconscious imagination and argue that appeal to it does not represent a departure from a standard view of imagination, before outlining my model and showing how it accommodates characteristic features of implicit bias. I argue for its advantages over the doxastic model: It does not violate the parsimony principle, it does not face any of the objections so far raised to doxasticism, and it can accommodate the heterogeneity with respect to structure in the category of implicit bias. Finally, I address whether my view limits our ability to hold people accountable for their biases (it does not), and whether it is consistent with what we know about intervention strategies (it is). I conclude that implicit biases are constituted by unconscious imaginings.

## 2 METHODOLOGY

Recent philosophy of mind attends to phenomena that do not fit neatly into our folk psychological categories, and responds in one of two ways. Sometimes philosophers propose new categories (e.g., Gendler's *alief,* 2008; Egan's *bimagination*, 2008). Sometimes they revise the most likely candidate category (e.g., Mandelbaum's *belief,* 2014). These strategies have their place, but only if we cannot explain the phenomena more parsimoniously. Better if we do not have to revise our folk understanding of cognition, or its components. This principle of parsimony is widely accepted.^1^

I will provide an account of implicit bias in line with this principle. I import a standard characterization of imagination, and have it tokened unconsciously. Although unconscious imagination occurs in nearby debates in philosophy of mind and aesthetics, I will not push my luck by presuming that this is a straightforwardly available resource. I will defend the claim that to appeal to unconscious imagination is not to adopt a revisionary notion of imagination.

I will contrast my model with Mandelbaum's doxastic model, largely leaving out of discussion models which characterize implicit biases as *patchy endorsements* (Levy, 2015), *aliefs* (Gendler, 2011), and *character traits* (Machery, 2016), among others. Levy's and Gendler's models do not honour the parsimony principle just sketched, and in addition I am persuaded by arguments against the existence of aliefs (e.g., Currie & Ichino, 2012; Mandelbaum, 2013). To put aside Edouard Machery's model I note that his view of traits is that they are constituted by mental states and dispositions (their so‐called *psychological basis*). If Machery's model of implicit bias were right, my position could be recast as one which described the psychological basis of implicit biases. More space is given to discussion of the unconscious belief model since it is most similar to my own, and it is important to get clear on the differences, and in virtue of what my view is to be preferred.

## 3 IMPLICIT BIAS

Jules Holroyd identifies the phenomenon of implicit bias as “the processes or states that have a distorting influence on behaviour and judgement, and are detected in experimental conditions with implicit measures” (Holroyd, 2016, p. 154). Implicit biases are fast and habitual^2^ and can operate in the absence of agent awareness (Holroyd, 2016, pp. 154–155) (later I discuss experimental results which suggest the possibility of awareness, Section 6.1). They are posited as items which cause common microbehaviours or microdiscriminations that cannot be tracked, predicted, or explained by explicit attitudes.

The term *implicit bias* has been used by some to refer to *biased behaviour* caused by an implicit attitude (e.g., Mandelbaum, 2016, p. 631), and by others to refer to a *mental item* which causes biased behaviour (e.g., Brownstein, 2016, p. 766; Levy, 2015, p. 800). If we understand implicit biases as mental items, the claim that they belong to a certain mental kind is coherent. If we understand implicit biases as biased behaviours, the interest is then in characterizing the mental items responsible for the biased behaviours. This is a merely terminological issue. Both frameworks recognize certain behaviours and mental items responsible for them. Whether we use *implicit bias* for one or the other is not a matter of substance. I will use *implicit bias* to refer to some mental item, rather than to biased behaviour, but what I argue can be put into either of these frameworks.

The canonical view of implicit biases is that they are associations whose existence can be traced to the learning history of the subject (Levy, 2015, p. 803). Concepts are associated with valences (e.g., *black male* with negative valence) such that in the presence of appropriate stimuli, these stored associations are activated. As Levy notes, though there is a good deal of variation between associative models, they agree on at least this much. More liberally, we might allow associations between concepts, for example between one's concept of *woman* and *weakness* (Mandelbaum, 2016, p. 630). On this view, an implicit bias is identified as the *association* between two mental items (see Fazio, 2007; Gawronski & Bodenhausen, 2011; Rydell & McConnell, 2006).

## 4 IMPLICIT BIASES AS UNCONSCIOUS BELIEFS

Recently there has been a move away from the associative picture to thinking of implicit biases as having propositional contents and not being involved in associative processes. If this is right, there is a specific relation between their constituents in virtue of this content, a relation which is absent if they are associations (Levy, 2015, p. 804). States with propositional contents can have satisfaction conditions, whereas states with only associative contents cannot (Mandelbaum, 2013, p. 199, fn. 1).

Mandelbaum cites a host of empirical evidence which he claims can only be accommodated by a propositional model of implicit biases (c.f., Toribio, 2018). For example, Gawronski, Walther and Blank's (2005) study showed that implicit attitudes follow the logic of “the enemy of my enemy is my friend.” Mandelbaum notes that “if you find two negatives making a positive, what you've found is a propositional, and not an associative, process” (Mandelbaum, 2016, p. 639). Further support for the claim that associative models are unable to account for many experimental findings is given by discussions of other work (for evidence that implicit biases are sensitive to argument strength, see Brinol, Petty, & McCaslin, 2009; for evidence that implicit biases are adjustable in light of peer judgement, see Sechrist & Stangor, 2001).

Mandelbaum's view is that implicit biases are unconscious beliefs.^3^ This view, he claims, can accommodate the survey of empirical results he presents as evidence against associative models.

### 4.1 Problems with the unconscious belief model

Although none of the objections considered here are decisive, I survey them to show the hurdles the view faces, which my imagination model does not (Section 6.3). Many problems have been raised which go via appeal to conditions on belief, followed by evidence that implicit biases fail to meet those conditions. These arguments take something like the following form: beliefs have feature *F,* implicit biases do not have feature *F,* therefore implicit biases are not beliefs.

First, implicit biases can be inconsistent with a subject's explicit attitudes. A meta‐analysis of data from Implicit Association Tests (IATs) put the correlation between implicit and explicit attitudes at 0.24 (Hoffmann, Gawronski, Gschwendner, Le, & Schmitt, 2005). Where implicit and explicit attitudes diverge, the doxastic model has to cast subjects as having inconsistent *beliefs.* 4 There is clearly a widely felt incoherence in supposing that the explicit egalitarian both does and does not believe that, for example, *women are weak.*

Second, Holroyd argues that harmony cases (in which one has implicit and explicit attitudes with the same content) are problematic for the doxasticist, since in order to characterize the implicit bias as a *belief* distinct from the explicit one, the claim that beliefs are individuated by their contents must be given up (Holroyd, 2016, p. 169).

Third, although Levy grants that implicit biases are propositionally structured, he claims that “their sensitivity and responsiveness to other mental representations is too patchy and fragmented for them to be properly considered beliefs” (Levy, 2015, p. 800). Levy argues that implicit biases often work non‐inferentially. For example, John F. Dovidio and colleagues' (1997) study showed that implicit race bias was predictive of certain behaviours when interacting with a black interviewer (i.e., less eye contact and more blinking) (Levy, 2015, p. 813). Levy argues that it is difficult to give an inferential account, in terms of belief, of what is going on here. In addition, a whole host of empirical evidence showing implicit attitudes' involvement in microbehaviours puts pressure on the view that implicit biases are beliefs (Levy, 2015, p. 813) (see Bessenoff & Sherman, 2000; Chen & Bargh, 1997; McConnell & Leibold, 2001; Wilson, Lindsay & Schooler, 2000). Levy also cites evidence that implicit attitudes do not behave like beliefs insofar as they do not update in the ways beliefs should in response to evidence (Levy, 2015, pp. 813–814). He draws on Rozin, Markwith and Ross's (1990) poison experiment, which he takes to demonstrate “the patchy and fragmented nature” of the content‐driven processing of the implicit attitudes involved (Levy, 2015, p. 815). In this experiment subjects were reluctant to take sugar from a jar labeled *not poison*, as compared with a jar labeled *safe*, even though they themselves affixed the labels. Levy offers this experiment as “one of a number of experiments that seem to demonstrate that non‐conscious processes are blind to negation” (Levy, 2015, p. 815). He takes this to speak against the unconscious belief model since blindness to negation represents “a pretty big chunk of the aptness for inferences associated with *bona fide* beliefs to go missing” (Levy, 2015, p. 815).

Similarly, Alex Madva argues that whilst implicit attitudes are insensitive to the logical form of an agent's thoughts, beliefs are not (indeed, sensitivity to logical form is a condition on belief) (Madva, 2016, p. 2661). Madva canvasses evidence that implicit biases respond to different logical form in similar ways (e.g., Gawronski, Deutsch, Mbirkou, Seibt, & Strack, 2008), and respond to the same logical form in different ways (Mendoza, Gollwitzer, & Amodio, 2010). In Gawronski and colleagues’ study, race bias was reduced when participants affirmed counterstereotypes (“YES” to for example, Black and *educated*), but *increased* when participants negated stereotypes (“NO” to for example, Black and *dishonest*) (Gawronski et al., 2008, p. 374).^5^ If affirming counterstereotypes (*black people are educated*) is more effective at reducing bias than negating stereotypes (*it is not true that black people are dishonest*), then though implicit biases are sensitive to *semantic content*, they are not sensitive to logical form. If sensitivity to logical form is requisite for belief status, implicit biases are not beliefs.

It is up for grabs whether the experiments on logical form are appropriate for influencing beliefs, and whether there are ways to explain the results in line with the doxastic model.^6^ In addition, none of the first three objections are decisive, particularly since Mandelbaum adopts a (version of a) Spinozan view of belief, defended elsewhere (Mandelbaum, 2014) according to which beliefs are formed as quickly as propositions are represented. This means that “the mere activation of a mentally represented truth‐apt proposition leads to immediately believing it” (Mandelbaum, 2014, p. 55). On this view just reading the words *dogs are made of paper*, caused you to form the belief that *dogs are made of paper* which, by now, you have likely revised. This unorthodox conception of belief may well be able to accommodate implicit biases having the features which seem to speak against a doxastic account of them.^7^ We might think, though, that claiming that some phenomenon belongs to a familiar category, like belief, is supposed to be explanatory in the sense that we can see why, in belonging to that category, it behaves as it does. We seem to lose that explanatory benefit if it turns out that the category is being thought of in a non‐standard way.

It might be objected that I cannot criticize Mandelbaum on the grounds that he imports a revisionary notion of belief, since my model imports a revisionary notion of imagination. However, as I make clear in the next section, my use of imagination is not a revisionary one. Nothing in our standard use of *imagination* rules out it being tokened unconsciously. And nothing I say about *unconscious imagination* in particular is in tension with how other folk have appealed to it in different explanatory contexts. Imagining tokened unconsciously is only a revision of a standard notion of imagining if one builds into that notion the claim that imaginings can only be tokened consciously. There is no principled reason to build in such a claim.

Let us take stock: We learn from Mandelbaum that there is evidence suggesting that implicit biases have propositional contents; we ought thus narrow the range of candidate mental states on which to model implicit biases. If belief is our only candidate we have good reason to discard the parsimony principle we began with and opt for a revised conception of belief. But objections abound for this view, as well as for the propositional nature of biases (e.g., Toribio, 2018). However, Mandelbaum is not motivated primarily by capturing implicit biases as some particular mental state; he notes that if the term *belief* offends, readers should feel free to understand his hypothesis as one about *structured thoughts* (Mandelbaum, 2016, p. 636).^8^ But if structured thoughts need not be identified as beliefs, and that is in keeping with the spirit of Mandelbaum's position, nothing has been shown beyond the propositional nature of biases (at best). The question of attitudinal nature remains open. Let us turn then to an alternative candidate.

## 5 UNCONSCIOUS IMAGINATION

Here I introduce the idea of unconscious imagination, before appealing to it in my model of implicit bias. I begin by noting features of imagination upon which there is “wide agreement” (Kind, 2016a, p. 1). First, imagination is a primitive mental state; it is irreducible to other mental states (c.f., Langland‐Hassan, 2012) (e.g., *perceiving, believing, remembering*). Second, it is a representational state, which is to say it is *about* something, it has intentional content. Third, and in contrast to perceiving and believing, imagining is not connected to truth (Kind, 2016a, pp. 1–3).^9^ These features are not exhaustive but are ones upon which there is wide agreement, and I note them here to signal my being fully signed up to such a standard conception. There is nothing revisionary here about my use of imagination. It is a state of this kind which, I argue, can be tokened unconsciously.

We can usefully distinguish two kinds of imagining: imagistic and propositional, where these kinds can have overlapping members (e.g., some propositional imaginings might involve mental imagery; Nanay, 2016a, p. 132, n. 1). If implicit biases involve unconscious imaginings, they may involve various kinds (I say more about this later, Section 6).

What then is *unconscious imagining?* By *unconscious* I mean *not available to introspection.* Thus, by *unconscious imagining* I mean a state with the above features which is tokened in such a way as to be not available to introspection. This is not to be confused with *non‐occurrent* imagining, understood in the sense used by Kendall Walton in cases of daydreaming.^10^ He gives the example of Fred, who daydreams about winning the lottery, the winnings of which are directed into a political campaign, which he wins, and then retires to the South of France. Of this case Walton claims that Fred has many non‐occurrent imaginings:

The question of whether his election is fair and square may never arise in his mind; he just takes for granted that it is, once he occurrently imagines returning to southern France, he has in the back of his mind the thought that his retirement is in a warm climate on the Mediterranean, even if he never gets around to saying this to himself. He thinks of himself, implicitly, as being in good health when he retires; he imagines that he is, but not occurently. These thoughts are, we might say, part of his “mental furniture” during the daydream. (Walton, 1990, p. 17)

Walton suggests that we ought not think of this case (and others like it) as a series of individual imaginings, one after the other. Rather, various imaginings are “woven together into a continuous cloth, although only some of the strands are visible on the surface at any particular spot” (Walton, 1990, p. 17). Of importance here, Walton claims that some non‐occurrent imaginings “are not necessarily unconscious ones”, and that we “may well be at least non‐occurently aware of them. (Perhaps noticing them occurrently would constitute imagining occurently)” (Walton, 1990, p. 18).

I will take the difference between non‐occurrent and unconscious imagining to come down to this: a non‐occurrent imagining is one for which, unsurprisingly, there is no answer to the question “when did the imagining occur?” An unconscious imagining is one which the subject is not directly aware of having, but which can occur at a particular time. I am interested in *unconscious occurrent imaginings.*

It might be thought that *unconscious* imagination represents a revision of our category of imagination, and so violates the parsimony principle with which I opened the paper. I will now argue that it does not. The three uncontroversial features of imagination I mentioned at the start of this section are neutral with respect to whether imaginings can be tokened unconsciously. Resistance to the possibility of unconscious imaginings will not be found in these uncontroversial features. I will now overview four reasons why it might be thought that imaginings cannot be tokened unconsciously. Each of these reasons proceeds by identifying some feature of imagination which cannot occur unconsciously. I argue that these additional features are not necessary to imagination, and so imaginings without them are imaginings nonetheless.

First, one might think imagining is *voluntary*, which makes it inappropriate to have it tokened unconsciously (in a way that it is not inappropriate to suppose belief could be tokened thus). However, although imagination is typically voluntary, it is not always:

Often we just find ourselves imagining certain things. Our fantasizing minds stray, seemingly at random, without conscious direction. Thoughts pop into our head unbidden. Imagining seems, in some cases, more *something that happens to us* than *something that we do.* Like breathing, imagining can be either deliberate or spontaneous. (Walton, 1990, p. 14, my emphasis)

Kind gives the example of being unable to stop imagining murder after seeing a gruesome murder in a horror movie (Kind, 2001, p. 91). She also notes, as does Walton, that we can find ourselves imagining, and be surprised at this. Several philosophers have appealed to involuntary imaginings in their work (e.g., Allen, 2015; Ichikawa, 2009), and though it may not be the most discussed case of imagining, it is imagining nonetheless. Unconscious imaginings are of this involuntary kind.

Second, imagining might be a mental *action,* and so a question arises about whether mental *actions* can occur unconsciously. The nature of mental action has not received much philosophical attention (Soteriou, 2009a, p. 1) relative to action simpliciter, and it is unclear what it takes for something to be a mental action, and thus what mental phenomena are candidates for this category. However, imagining is often put forward as a candidate for mental action (e.g., Hieronymi, 2009, p. 146; Proust, 2009, p. 266; Soteriou, 2009b, p. 233), and so it might well be asked whether *unconscious* imaginings are properly described in these terms.

Unconscious imaginings are not mental actions, but that does not mean that including them in our category of imagination requires revision of that category. If *conscious imaginings* are indeed mental actions, there is no reason to suppose that this is *qua* being an *imagining.* Plausibly, there is an accessibility requirement on something's being a mental action (O'Brien, 2009; Soteriou, 2009a, 2009b), which rules out unconscious imaginings as members of this category. That would only be a problem if one thought all imaginings are mental actions. Imagining simpliciter need not be a mental action, and at least some of those who refer to imagining as an example of a mental action are clear about this. (Soteriou (2009b) claims that “kinds of imagining” are mental activities, p. 233, while Hieronymi (2009) claims that unintentional imaginings “need not be treated as instances of mental agency” [p. 146, n. 13], where mental agency is a category which includes mental actions.)

Third, one might worry not about imaginings being *voluntary* or *mental actions* but that they are *subject to the will.* I am not persuaded that all imaginings are subject to the will (see Allen, 2015, p. 295, for discussion), but in any case, presumably the thought is that imaginings one is *directly aware of* are, at least in principle, subject to the will. Unconscious imaginings fall outside the scope of this claim. What about if one took *being subject to the will* to be a necessary condition on imagination? Then one might reason to the denial of the possibility of unconscious imagination, or to the inclusion of unconscious imaginings in our category of imagination representing a move away from more standard notions of imagination. However, given the evidence that some strategies are successful in changing or extinguishing our implicit biases (Section 7), if implicit biases involve unconscious imaginings, they may well be subject to the will, at least sometimes, and in at least some conditions.

Fourth, it might be thought that making imaginings unconscious represents a revision of our standard notion of imagination because all imagining involves *conscious imagery,* and so there is a contradiction lurking in the very idea of an *unconscious imagining.* As with the first three points, nothing in the three uncontroversial features I noted earlier supports this constraint on imagination. In particular, the second uncontroversial feature of imagination (that it has representational content) does not place any restrictions on the kind of representational content imaginings can involve. That said, I make two points in reply to the claim that all imaginings involve conscious imagery: First, not all imaginings involve *imagery,* and second, not all imagery is *conscious.*

First then, the theoretical standing of the view that imagery is necessary to imaginings (*image essentialism*) cannot be settled here, and though some have taken it to be obvious that it is not (e.g., Ryle, 1949; Walton, 1990; Yablo, 1993),^11^ others have defended the claim that it is (e.g., Kind, 2001) (for discussion, see Gregory, 2016). I am on the side of those who recognize imagining without imagery. However, if image essentialism were true, the details of my account would require revision, not rejection. As I will argue later, both imagistic and propositional imaginings ought to be in our model of implicit bias. Importantly, the image essentialist does not deny that imaginings can have propositional content, she only insists that that content is accompanied by mental imagery. At worst, it would need only be said that in cases of propositionally structured unconscious imagining, there is accompanying imagery. So long as it were not also the case that imaginings, insofar as they must involve imagery, can only be tokened consciously, then we are free to speak of unconscious imaginings, even in the context of restraints on content imposed by image essentialism.

To my second point, not all imagery is conscious. Some have thought that unconscious perception naturally paves the way for unconscious imagery:

[I]f perception can be conscious or unconscious, then it is difficult to see what would prevent one from having both conscious and unconscious mental imagery. (Nanay, 2013, p. 105, see also Spaulding, 2016a, p. 210)

The possibility of unconscious perception does not *entail* the possibility of unconscious imagery—one may very well accept one and not the other. Note also that some sceptical of unconscious perception nevertheless allow for the possibility of unconscious imagery (Phillips, 2016, 2014, respectively). Thus the case for unconscious imagery does not *rest* on unconscious perception, but may well be supported by it.

In any case, there is no good reason for denying the possibility of unconscious imagery, and so no good reason for its inclusion in the possible contents of imaginings to be taken as revisionary. For now, then, I refer the reader to discussion elsewhere in support of it (Church, 2008, pp. 292–294; Nanay, 2010, 2013,12 2016a, Phillips, 2014, Spaulding, 2016a, p. 210) and place the burden on she who wants to deny it.

Note that only the *combination* of the worries that imagining necessarily involves imagery and that imagery is necessarily consciously tokened suggests that unconscious imagination is a revisionary notion. One can have image essentialism providing unconscious mental imagery is countenanced, and one can have the claim that imagery can only be tokened consciously providing purely propositional imaginings can be countenanced. Though both tolerable, as will become clear, such views of imagination would require revision of my model or its intended scope.

Having responded to four routes to the claim that unconscious imagination represents a revision of our standard notion, it is worth noting that unconscious imagination has recently gained some currency in the philosophical literature. Jennifer Church defends and appeals to the notion to give an account of three cases^13^ which she argues cannot be explained without appeal to unconscious imagining (Church, 2008, see also her work on perception, 2013, ch. 2, 2016). Van Leeuwen (2011) notes that not all forms of imagination he posits are “necessarily part of *conscious* experience” (p. 57, fn. 4). Goldman (2006) introduces the term *enactment‐imagination* in his simulation account, noting that the process of enactment‐imagining, and the products thereof, can either be “conscious or covert” (p. 151). In a similar vein, Spaulding (2016b) appeals to unconscious imagining in her account of low‐level simulational mindreading (pp. 268–71).^14^

I am not committed to an explanation citing unconscious imaginings being correct for all or any of these cases; perhaps they are better explained in a way which does not involve unconscious imagining.^15^ I refer to them only to gesture at the work unconscious imagining has been claimed able to do, and to motivate the legitimacy of appealing to it elsewhere. Unconscious imaginings have been given less attention than their conscious counterparts, and although it might be surprising to think of imagination tokened unconsciously, I suspect that this is a function of its being relatively underrepresented in work on imagination. Imagination tokened unconsciously does not represent any move away from the uncontroversial characterization of imagination I began with.

The goal of this section was to make the reader more willing to recognise that unconscious imagining does not mark any departure from standard notions of imagination. The claims that all imaginings are voluntary, mental actions, subject to the will, or involve conscious imagery, are ones which go far beyond the widely agreed upon features of imagination we began with. A notion of imagination which honours the three standard features but does not honour these additional claims to which some theorists are attracted is not, therefore, a revisionary one. Recognition of unconscious imaginings is especially important if an account of implicit bias which appeals to it comes with explanatory riches. It is the task of the work which follows to show that unconscious imaginings earn their keep in this context.^16^

## 6 BIASED BY OUR IMAGININGS

My view is that implicit biases are constituted by unconscious imaginings. I will argue that this view has the explanatory credentials won by accommodating the primary features of biases, and does not face the problems of the doxastic model. This is in part down to the heterogeneity present in the class of *implicit bias,* and that in the class of *imagination.* An additional attraction of the imagination model is that it is uniquely able to accommodate the heterogeneity of implicit biases with respect to their structure.^17^ In particular, it can accommodate implicit biases being structured associatively (i.e., multiple imaginings) or non‐associatively (i.e., single imaginings). That this is so is not the result of strategic imprecision so as to protect the thesis from falsification,^18^ but is rather motivated by the need for the model to be extensionally adequate. If there is heterogeneity along this dimension (as suggested by empirical work) the imagination model can accommodate it.^19^ Other models cannot, since the type of state they identify is determinately propositional or not. I will run through an example of implicit gender bias to demonstrate this.

To have an implicit bias is to unconsciously imagine certain things in response to stimuli. Let us turn first to capturing implicit biases as involving associative processing. In this framework, one of three things could be going on. First, in the presence of a woman‐stimulus, a subject has an unconscious imagistic imagining of a *woman* which is associatively linked to an unconscious imagistic imagining of *weakness.* Second, a subject has an unconscious imagining of a *woman* which is associated with a negative valence (it is in these two ways of understanding what is going on that the imagination model is most similar to an associative model where the things associated are concepts with other concepts, or concepts with valences). Third, incorporating Mandelbaum's (2016) insight that there can be associative transitions between propositions (p. 633), being presented with a woman‐stimulus may cause a subject to have an unconscious propositional imagining with the content *there is a woman* associatively linked to an unconscious propositional imagining with the content *there is weakness.* On these ways of understanding implicit bias, the constituents of the bias are associatively linked, and do not stand in determinate syntactic relations.

But what about the evidence which suggests that implicit biases are not involved in associative processes, but rather the bias's constituents are held as standing in a determinate relation? There are two ways the imagination model can capture what is going on here which does not involve associative processes. First, when presented with a woman‐stimulus, one could imagistically unconsciously imagine a *weak woman.* In this case, the implicit bias is identical to a single instance of unconscious imagistic imagining, rather than being the association between two unconscious mental images. Alternatively, one could unconsciously propositionally imagine that *women are weak* (it is here that the imagination model and belief model are very similar), in such a case the contents of the proposition are held to stand in a determinate relation.

I have demonstrated the ways in which implicit biases can be understood as constituted by unconscious imaginings which honour the heterogeneity within this category with respect to their structure. Further work might allow for finer carving of the category of *implicit bias*, which would naturally facilitate our delineating the rather different roles that unconscious imaginings play.

I now turn to showing how my model can accommodate the paradigmatic features of implicit bias that we started with: our being *unaware* of our implicit biases and them having downstream effects on judgements and behaviour.

### 6.1 Awareness

Implicit biases are attitudes of which we are not aware, at least in ordinary circumstances, and they operate fast and habitually. Their operating in this way is consistent with them being constituted by unconscious imaginings taking part in associative or non‐associative processing. There is nothing about the nature of *unconscious imagination* which rules it out from operating fast and habitually.

Our being unaware of our biases is one of their “remarkable features”; neither “introspection nor honest self‐report are reliable guides to the presence of such mental states” (Kelly & Roedder, 2008, p. 532). This coheres with a model of these states as unconscious imaginings: given that these imaginings are unconscious (i.e., not available to introspection), our not being aware of them is as expected.

A complication with the general claim that we are unaware of biases is that sometimes we are able to become aware of them, or at least, we are able to estimate (with some accuracy) the biases we have. Demonstrations of this come from so‐called *bogus‐pipelines* studies. For example, Jason Nier investigated the relationship between implicit racial attitudes and self‐report scores on the Modern Racism Scale (MRS), finding that:

when participants believed their ‘true attitudes’ were being accurately assessed, there was a significant relationship between an implicit measure of racial attitudes (the IAT) and an explicit measure of racial attitudes (the MRS). When participants did not believe that their self‐reported explicit attitudes could be accurately corroborated with an implicit measure, there was no association between implicit and explicit attitudes. (Nier, 2001, pp. 48–49)

How does my view explain results like this? A clue comes from studies on predicting implicit attitudes. Adam Hahn and colleagues found that participants were able to predict their IAT results, even when their explicit attitudes did not coincide with their implicit ones. They hypothesize that participants were accurate in their predictions because when they “were presented with the attitude targets, participants did in fact ‘feel’ their affective reaction and reported on those reactions as their implicit attitudes, even though they might have invalidated those same responses as a basis for their explicit attitudes” (Hahn, Judd, Hirsh, & Blair, 2014, p. 1387). Nier's participants who believed their “true attitudes” were being accurately assessed may have allowed themselves to be guided by their affective reactions when reporting their explicit attitudes. Ranganath and colleagues draw similar conclusions following their study on the correlation between self‐reported “actual feelings” towards straight/gay people and implicit attitudes, as compared to the correlation between self‐reported “gut feelings” about straight/gay people and implicit attitudes. *Actual feelings* were understood to be those participants experienced when given time for consideration, whilst *gut feelings* were those experienced when initially thinking about the attitude target (Ranganath, Tucker Smith, & Nosek, 2008, p. 388). Reports of gut feelings were more negative than reports of actual feelings and more closely correlated with implicit attitudes. Ranganath and colleagues conclude that:

By having participants focus on their gut feelings, it is possible that participants are referring to aspects of their cognitive or affective experience that are more related to automatic processes than they would when responding to a typical self‐report measure. (Ranganath et al., 2008, p. 393)

Imaginings are well‐placed to produce these cognitive or affective experiences.^20^ We can become nervous when we imagine visiting the dentist next week, scared when we imagine a china doll under the bed, excited when we imagine meeting our favourite politician at a rally. If Hahn and colleagues are correct about the mechanism via which we can make good predictions about our biases, my imagination model can accommodate this.^21^

Do we need to be aware of the content of our imaginings for them to produce cognitive and affective experiences? If we did then unconscious imaginings could not produce the cognitive and affective experiences characteristic of implicit bias. However, there is no reason to think that awareness is required. The discussion takes place in the context of a hypothesis which attributes to mental items causal powers in the absence of content awareness. If biases can have downstream effects on cognition and affect when we, *ex hypothesi*, do not have content awareness of them, but infer it from our responses to stimuli, why can we not suppose that imaginings also do? For those who think there is a principled difference lurking, the burden is on them to demonstrate this.

### 6.2 Effects on judgement and behaviour

There is substantial evidence that implicit biases affect judgement and behaviour.^22^ For example, subjects are slower to press a button which associates positive words with black faces, than a button which associates positive words with white faces. In a video game, people are faster to “shoot” an armed subject if he is black than if he is white, and faster to “not shoot” an unarmed subject if he is white, than if he is black (Correll, Park, Judd, & Wittenbrink, 2002). People more quickly identify guns when primed with a black face as compared to when primed with a white face (Payne, 2001), and when a time constraint is introduced, participants more often mistakenly identify tools as guns when they are primed with a black face as compared to when they are primed with a white face. People select CVs with male names over identical CVs with female names (Steinpreis, Anders, & Ritzke, 1999). Also, measures of implicit bias can be predictive of future behaviour. For example, IAT results can predict—better than explicit attitudes—subtle racist behaviours (McConnell & Leibold, 2001).^23^

How does this feature of implicit bias get explained on my model? If the implicit bias in play is underpinned by associative processing (where the components are imagistic or propositional imaginings), the constituents of the associative process are not where the motivational power lies. In these cases, a standard associative explanation can be given. In the presence of a black face, a subject has associatively linked unconscious imaginings. The unconscious imagining of *black man* activates the unconscious imagining of *danger,* which makes danger‐related concepts more accessible (Levy, 2015, p. 805).

In cases of non‐associative bias involving single imaginings, the imagining itself needs to be the kind of thing with motivational credentials. Imaginings can guide behaviour,^24^ though this guidance is limited (in a way that the behaviour‐guiding role of belief is not, cf. Ichino 2015) (Glüer & Wikforss, 2013, pp. 143–145; Noordhof, 2001, p. 253; O'Brien, 2005, p. 59). Examples include childhood pretense,^25^ checking in one's wardrobe for monsters after seeing a horror film, and so on. If implicit biases are imaginings, this would capture the downstream effects on behaviour and judgement characteristic of implicit bias.

As for the mechanism, it can be understood as the same kind of mechanism as other non‐associative accounts. The idea is that when presented with a black face, a subject with a propositionally structured implicit bias, or a single imagistic imagining, takes there to be a *determinate relation* between *black man* and *danger* (i.e., the constituents of her attitude) (Levy, 2015, p. 805). So when subjects are slower to pair black faces with positive adjectives, the imagination model can explain this by appeal to imaginings positing determinate relations between the target stimuli and some feature.

These various mechanisms can explain the results of Correll and colleagues' (2002) shooter task, Payne's (2001) affective priming task, why one is more likely to perceive ambiguously aggressive behaviour as hostile when carried out by a black person, rather than a white person (see, e.g., Duncan, 1976; Sagar & Schofield, 1980), and why one is likely to undervalue a CV headed by a female or foreign name, as compared to one headed by a male or non‐foreign name. On my model the hypothesis is that when primed with certain stimuli (black person, or female name), one makes judgements or engages in behaviours convergent with what one is occurrently unconsciously imagining.

Is awareness of content required for behaviour guidance? Again, we are operating in the context of a discussion which asks how mental items of which we do not have content awareness can have the functional profile they do with respect to judgement and behaviour. There is no reason to think that imaginings—the kinds of state which can motivate judgment and behaviour—can only do so when their contents are ones of which we are aware. *Ex hypothesi*, whatever these behaviours are guided by does not require content awareness. Specifying the contents of these states might be explanatory, but not in virtue of those contents being introspectively available to the subject.

Relating to this is the following observation: imaginings of which we are aware may affect our behaviour in a different way to the way implicit biases affect our behaviour. For example, imagining a monster under my bed makes me check under my bed before sleep. In this case if asked why I behaved as I did I may give the reason (which I recognize as silly), and I do not confabulate reasons. On the other hand, when I give a low grade to a piece of work with a female name on it, or cross the road to avoid a black man, I am not aware of the content of the attitude which guides me. When asked about my behaviour, I may well confabulate reasons for it when I say things like “the essay was poor” or “that side of the street was busy” (see Sullivan‐Bissett, 2015). We can make sense of this behaviour on any model which casts implicit biases as motivating, and as unconscious. So although imaginings we are conscious of may guide behaviour differently and content awareness of these imaginings allows for true behavioural explanations, imaginings *simpliciter* are nonetheless candidates for behaviour guidance, even if lack of content awareness makes the guidance different, and behavioural explanations less reliable.

The imagination model accommodates different constitutions of implicit biases, which may help explain different influences on behaviour (see Holroyd & Sweetman, 2016, pp. 91–92 for discussion). As above, the mechanisms for behaviour guidance by an implicit bias will differ depending on whether the unconscious imagining(s) in play is part of an associative or non‐associative process.

### 6.3 Advantages over the doxastic model

Here I return to the objections raised earlier to the doxastic model and show that the imagination model does not face them.

First, there is no problem with accommodating inconsistent content of implicit attitudes and explicit attitudes on my model. As we saw earlier, on Mandelbaum's view in inconsistency cases the subject has inconsistent beliefs. I noted that there is a widely felt incoherence in supposing that the explicit egalitarian both does and does not believe that, for example, *women are weak.* There is no such incoherence on my model. There is no constraint on imagination that one should, or can only imagine contents in line with what one believes; indeed, this is one of the features of imagination taken to have universal appeal (Kind, 2016a, p. 3). That the content of some implicit biases is not aligned with explicit attitudes is seamlessly accommodated by the imagination model.

Second, Holroyd objected to the doxastic model by appeal to harmony cases. The problem, recall, was that in order to characterize implicit attitudes as beliefs distinct from explicit ones, the claim that beliefs are individuated by their contents must be surrendered (Holroyd, 2016, p. 169). My model does not face this problem since alignment cases are not alignment of two beliefs, but rather, at worst (in cases involving a single unconscious imagination), alignment of a single unconscious imagining and an explicit belief.

Third, Levy was concerned by studies which show that implicit biases do not partake in inference, or, at least, that it would be difficult to tell a story about certain behaviours via appeal to unconscious beliefs. Not all of the ways imaginings underpin implicit bias need be supposed able to enter into inferential processing (i.e., those involved in associative processing), and so in cases where it is difficult to give an inferential account, we can appeal to imaginings involved in associative transitions. In such cases the model does better not by appeal to the nature of imagination, but in virtue of its being inclusive of both associatively and non‐associatively structured implicit biases. Nevertheless, some studies (those Mandlebaum cites) seem to show that implicit biases partake in inference, and the imagination model can accommodate those cases too. Imaginings can stand in inferential relations: if I imagine that *my house is on fire*, and imagine that *my house being on fire puts me in danger*, I will also imagine that *I am in danger* (Sinhahabu, 2013, pp. 160–161). In those cases in which inference seems to occur, the imagination model has the tools to accommodate them.

Finally, turning to the objection from Madva (and similarly Levy, 2015, pp. 813–814), that beliefs are sensitive to logical form, but implicit biases are not. Imagination is sometimes insensitive to logical form. In support of this, let us turn to Currie and Ichino's response to Gendler's criticism of imagination‐based explanations of Rozin and colleagues' (1990) cases:

Notably, in the “Not sodium cyanide, not poison” case, the label also bore a skull and crossbones image preceded by the word “Not”. An erotically charged image preceded by the word “Not” is unlikely to provoke images of celibacy. (Currie & Ichino, 2012, p. 792)

That is, imagination can be sensitive to semantic content, but insensitive to logical form, in particular, we should expect that in some conditions these attitudes respond to differing logical form in similar ways. The involuntariness of some imaginings demonstrates this: having seen a horror movie, a friend telling you that there *is not a monster in the wardrobe,* will hardly help you to stop imagining that there is (though it ought to stop you believing that there is). Or if I tell you that there is *not a zebra under the table,* that may well instill in you an imagining that there is, though it will not cause you to form the corresponding belief (as noted earlier; fn. 7, even on the Spinozan model we should not expect beliefs to be insensitive to logical form). Of course, some imaginings may be sensitive to logical form. If I learn that my colleague is not in her office today, that may well stop me imagining that she is when I plan to speak to her later. For the imagination model to accommodate the insensitivity to logical form which has been argued to be a feature of some implicit biases, it need only be the case that some imaginings are so insensitive.

Even if the doxastic model can answer all of these objections, two issues remain. First, in its appeal to a revisionary notion of belief it violates the parsimony principle set out earlier. I argued that although unconscious imagination has not captured the attention of many imagination theorists, it does not require a revision of the basic notion of imagination. If the reader is not convinced by this point there nevertheless remains a second issue for the doxastic model: it cannot accommodate the heterogeneity of implicit biases with respect to their being involved in associative and non‐associative processes.

At this point it might be wondered whether a view on which implicit biases are different kinds of unconscious imaginings associatively linked (or not) is preferable to one that divided them up between two other different kinds of mental states (to accommodate the same evidence). However, there is no such view yet in the offing. Until there is, my view which can accommodate the heterogeneity of biases is in good standing. In addition, it might be that different kinds of imagining could explain why even biases against the same social group as measured by different indirect measures, or even the same measures testing for different biases against the same group, are not correlative (see Machery, 2016, p. 116, and Amodio & Devine, 2006, respectively). For example, implicit racism scores on the IAT are poorly correlated with scores on Payne's weapon task (Mandelbaum, 2016, p. 631). This might be because these cases involve imaginings with different contents, or imaginings taking part in different processes, such that one kind of bias can be present without the other in some subjects. (See also Del Pinal & Spaulding, 2018, for a hypothesis about encoding which accommodates what looks like bias loss across context change.)

## 7 MORAL STATUS AND INTERVENTION

I have argued that my model can accommodate the features of bias I outlined at the start of the paper, as well as the heterogeneity present in this psychological category. Before closing, I speak to two more issues. The first is whether my view will limit our ability to hold people accountable for their biases on the grounds of them being morally problematic. The second is whether my view is consistent with the data on intervention strategies.

To the first task: we might take it as a datum that at least some implicit biases are morally problematic. If implicit biases are constituted by unconscious imaginings, is this result delivered? I make two points about this. First, many models of implicit bias do not pave an obvious road to particular moral judgements (i.e., models on which implicit biases are associations, patchy endorsements, or aliefs). It might be thought that the doxastic model gains an advantage here, but in fact, it does not. Given the view of belief adopted by Mandelbaum (one of automatic formation on which we believe any truth‐apt proposition we represent), it is very much up for grabs whether beliefs are candidates for inclusion in the moral domain. In cases of conscious belief we could hold folk accountable for not being scrupulous enough to revise or reject their automatically formed belief, but it is far from obvious that this way of retaining moral responsibility for our beliefs is available in cases of unconscious belief. Second, if the moral judgements we make about implicit biases are based on what we know about the likely effects of those biases, then many models do equally well. This is because what we are up to is condemning the *judgements* and *actions* which are downstream effects of biases, and there is no reason we cannot tell the same story about any candidate mental state which can have these downstream effects, such as imagination.^26^

To the second task: The imagination model also accommodates empirical work on intervention. Here I will briefly describe some empirical work identifying which techniques can lead to the reduction of bias. I will then suggest that implicit biases being constituted by unconscious imaginings is a good explanation of these results.

Irene Blair and colleagues investigated the effect mental imagery had on moderating implicit bias. They describe *mental imagery* as “the conscious and intentional act of creating a representation of a person, object, or event by seeing it with the ‘mind's eye’” (Blair, Ma, & Lenton, 2001, p. 828). In one of five experiments, participants were either in the counterstereotype group, or the neutral (control) group. In the first, participants were asked to imagine a strong woman, in the second, participants were asked to imagine a holiday. Blair and colleagues found that those in the counterstereotype group “produced a significantly lower level of the implicit stereotype than the participants who imagined a neutral event” (Blair et al., 2001, p. 831). In discussion they say that imagining a counterstereotypical exemplar “reduced the implicit stereotype by more than half, providing the first demonstration that mental imagery can have a powerful effect on implicit processes” (Blair et al., 2001, p. 831).

In another experiment, Peck and colleagues were interested in whether body representation can change implicit attitudes, that is, in whether, to reproduce the title of their paper, “putting yourself in the skin of a black avatar reduced implicit racial bias” (Peck, Seinfeld, Aglioti, & Slater, 2013). Experimental participants wore a head‐mounted display, through which they would see a programmed virtual body which substituted their real body. A body‐tracking suit was also worn, which meant that when they moved their real body, their virtual body would move in the same way. Peck and colleagues found that those participants who were embodied in dark skinned bodies had less implicit race bias after the exposure, as compared with those embodied in light skin bodies, and those not embodied at all.

In their discussion of the experimental results, Peck and colleagues suggest that by embodying a participant in a dark skinned avatar, they were able to “temporarily transfer someone to a different in‐group”, which “could be argued to be a very powerful way of transforming group affiliation” (Peck et al., 2013, p. 785). (See also Johnson, Jasper, Griffin, & Huffman, 2013 and Young, 2017 for perspective taking decreasing bias.)

The two experiments just overviewed show that imagining countersterotypical examples, or being embedded into a target group member's body, can help combat implicit biases and mitigate their effects. These results are easily accommodated by the imagination model. We can be caused to imagine all sorts of things by the sexist, racist, and heteronormative culture many of us inhabit (Dasgupta, 2013, p. 240). When engaging in imaginative activities, like imagining a countersterotypical exemplar, the existence of, or effects of, these implicit biases change. Indeed, Blair and colleagues note in their explication of mental imagery that it increases the “accessibility of related cognitive, emotional, and behavioural representations” (Blair et al., 2001, p. 829). In its doing so, it could make less accessible opposing cognitive, emotional, and behavioural representations in line with the unconscious imaginings, that is, in line with the targeted implicit bias. (See also Dasgupta & Greenwald, 2001 for evidence of decreased race bias after exposure to respected African Americans and disliked white people. I take it a similar mechanism is involved here, with bias decreasing as a result of counterstereotypical stimuli increasing the accessibility of related representations.)

Next consider the results from Peck and colleagues' (2013) study. When a subject is transformed into a dark skinned avatar, that subject may find themselves imagining (consciously or unconsciously) that they have dark skin, that they are a member of a different racial group. Again, with these kinds of imagining, it may well become more difficult to have unconscious imaginings constitutive of implicit race bias.

The imagination model can accommodate empirical findings regarding the effects of certain interventions on bias, and it can also speak to why such effects are short‐lived. As Huebner (2016) points out, strategies for impacting on the expression of implicit biases are limited, since they depend on relatively local interventions, which take place in experimental conditions, isolated from the kinds of inputs which shape our biases. However, once participants leave such conditions, they are once again assaulted by the kinds of influences which gave rise to their biases in the first place. As Dasgupta notes, “individuals' implicit attitudes will reflect whatever local environments they are chronically immersed in” (Dasgupta, 2013, p. 271). This is supported by the fact that *repeated exposure* to non‐stereotypical properties of a group can influence our biases (Huebner, 2016, p. 70). I suggest that we are caused to imagine all sorts of things as a result of the environment we are immersed in, which, unfortunately, is all too often powerful enough to undo the good work of intervention strategies practiced in experimental conditions.

## 8 CONCLUSIONS

Consider psychological category *x. x* has members which are associatively linked, as well as members which are propositionally structured and do not enter into associative processing. Its members are tokened unconsciously but occurrently, they guide some judgements and behaviours, produce affect, and are tokened habitually and involuntarily. How should we understand this category? The literature on implicit bias labels its members *implicit bias.* I have argued here that the functional profile of implicit biases can be captured on a model which appeals to unconscious imagination. Implicit biases being constituted by unconscious imaginings coheres with our understanding both of biases, and with the functional role of unconscious imaginings in cognition.

Thus implicit biases—which we do not know what to make of—are in fact constituted by unconscious imaginings. This is a category which we have not attended to much, but which is a legitimate and non‐revisionary combination of two things we know a lot about; the unconscious, and the imagination. I have shown that my account can accommodate key features of implicit bias, as well as heterogeneity in this class along two dimensions, and thus I suggest that implicit biases are constituted by unconscious imaginings.

## Footnotes

1 For example, Levy (2016) claims that “we ought to avoid multiplying mental states unnecessarily, we need to ensure that we are postulating exotic states and processes only when they are truly needed” (p. 9). See also remarks from Egan (2011, p. 68), Anna Ichino (2015, p. 19), and Jake Quilty‐Dunn (2015, p. 277).

2 Holroyd (2016) describes the operation of biases as “fast and automatic” (p. 154). However, given that implicit biases are sensitive to context (see, e.g., Govan & Williams, 2004; Wittenbrink, Judd, & Park, 2001), it is more accurate to describe them as “habitual”.

3 Strictly speaking, Mandelbaum's view is that the implicit attitudes guiding biased behaviour are unconscious beliefs. Keith Frankish also argues that implicit biases are the effects of implicit beliefs. He distinguishes *implicit* beliefs (those which have an effect on thought and behaviour without conscious recall) and *explicit* beliefs (those whose downstream effects require conscious recall) (Frankish, 2016).

4 The doxasticist may well appeal to fragmentation in light of this. Mandelbaum (2016) has argued that our belief systems are fragmented, where some beliefs are active in some contexts, and other beliefs are active in others (p. 650).

5 Though as Madva notes, Gawronski and colleagues did not test the effects of affirming stereotypes or negating counterstereotypes, a comparison of these is needed for a direct test of biases interacting with the same logical form in different ways (Madva, 2016, p. 2669).

6 See for example Johnson, Kopp and Petty (2018) for criticisms of the studies purporting to show logical form insensitivity, and Mandelbaum's first comment on the Brains Blog symposium on Del Pinal and Spaulding's (2018) paper.

7 Although this unorthodox conception of belief does not help in the face of Madva's objection (it is no part of the Spinzoan model that beliefs are insensitive to logical form).

8 It is due to this feature of Mandelbaum's position that I have foregone discussion of the plausibility of the Spinozan model of belief (see Sperber et al., 2010, pp. 362–364, 368–369 for discussion of Gilbert, Krull, & Malone's, 1990 and Gilbert, Tafarodi, & Malone's, 1993 empirical work in support of the Spinozan model). Although Mandelbaum is wedded to such a model in his other work (Mandelbaum, 2010, 2014; Mandelbaum & Quilty‐Dunn, 2015), his primary aim in articulating a model of implicit bias is to show that such mental items are propositionally structured. He takes them to be (Spinozan) beliefs, but from his point of view, those not already signed up to the Spinozan model are welcome to resist that characterization.

9 Kind understands this third feature as specifying the absence of a *constitutive* connection to truth. I have dropped the “constitutively” here since I think belief's connection to truth is a contingent one (Sullivan‐Bissett, 2017; 2018). Those who want the stronger claim would sign up to the weaker claim as I have it here: belief is connected to truth in a way that imagining is not. For discussion of the difference between belief and imagining in this context see Sinhahabu (2016), pp. 120–121. In addition, to say that imaginings are not connected to or constrained by truth is not to deny that they have some such relationship (they are, for example, states with truth‐evaluable contents). What they lack which belief does not is to be understood in a more heavy‐duty sense, often captured by the slogan that belief *aims at truth.* Whatever imagination's relationship to truth is, it is not one of being so aimed.

10
*Occurrent* has been contrasted with *dispositional* in the discussion of occurrent beliefs, understood as the status beliefs have when at the forefront of one's mind (Schwitzgebel, 2015). Here I use the term to pick out whether there is a time at which an imagining *occurred.*

11 In addition, some people report *no imagery* but do not suffer from an inability to *imagine* (Faw, 2009).

12 Nanay gives something like my view in his discussion of pragmatic mental imagery. Of the avowed anti‐racist with implicit race bias he suggests that the subject “forms different pragmatic mental imagery in response to seeing an image of a Caucasian and an African face” (Nanay, 2013, p. 126), and he takes the difference here to explain the behaviours the subject exhibits in the presence of Caucasian and African faces. There are a couple of differences between our views worth noting. First, Nanay thinks implicit biases are identical to unconscious *imagery* which need not be understood as unconscious *imagination* (Nanay, personal communication, May 31, 2018). Second, he does not seek to accommodate propositionally structured implicit biases in his model.

13 Here is one such case: “A mother goes into her child's room and looks through the child's things while the child is away […] As she searches the room, she steps softly and keeps her face angled toward the door; her body is visibly tense. Her movements are not those of an officer investigating the scene of a crime so much as those of a stealthy burglar” (Church, 2008, p. 380). Church argues that the mother does not *believe* someone might be in the house, nor does she *consciously imagine* it. She acts *as if* someone might be in the house. Church claims that we cannot explain the mother's behaviour by appeal to her conscious mental states. Instead it is an unconscious imagining that *someone is in the house* which makes the mother act as she does.

14 An additional proponent of unconscious imagination might be Nanay. Kind argues that the plausibility of Nanay's (2016b) model of decision making “hinges on our acceptance of unconscious imagining” (Kind, 2016b, p. 2), though Nanay does not explicitly commit himself to this. Kind takes it that appealing to unconscious imagining is under‐motivated or ad hoc (Kind, 2016b). If my model of bias is right, such a worry loses its force (at least insofar as explanatory riches speak to our ontological commitments).

15 For example, in Church's cases we might appeal to *acceptance in a context* (Bratman, 1992).

16 Even if one thought that what I am describing are not *imaginings*, but something just like imaginings except only for being unconscious, a state like that would suffice for me. However, I see no reason to further carve up the conceptual space in response to mere pre‐theoretical resistance to recognizing imaginings tokened unconsciously. This would also be an approach in possible violation of the parsimony principle I began with.

17 Other theorists have sought to accommodate heterogeneity in the class of implicit bias, though none along the lines of whether they are involved in associative or non‐associative processing. Machery's claim to heterogeneity is a function of his being non‐committal with respect to the attitude involved in implicit bias, since he thinks implicit biases are traits, multiply realizable by different psychological bases. However, insofar as Machery (2016) speaks to the question regarding the structural nature of bias, he has it that one component of the psychological basis of implicit bias is an *association* between concepts (p. 112). Bryce Huebner's claim to heterogeneity is based on his view that implicit biases reflect the combined influence of three computationally and psychologically distinctive evaluative systems (associative processing, associative Pavlovian systems, and associative model‐free systems) (Huebner, 2016, p. 51). But again, insofar as he speaks to whether biases are associatively structured, he is firmly on the side of the associationists, and interested in how these associations get internalized. Finally, Del Pinal and Spaulding speak to the heterogeneity of biases, though at the level of their encoding. They argue that although some biases may be encoded as salient‐statistical associations, others are encoded “in the dependency networks which are part of our representations of social categories” (Del Pinal & Spaulding, 2018, p. 96). This means that concepts can encode information regarding cue‐validity and saliency, but also the degree of centrality of their associated features. However, Mandelbaum has argued that it is difficult to understand their proposal without talk of the vehicle of the encoding (i.e., whether we are talking about associations or propositional states) (Mandelbaum, 2018, p. 2).

18 The flexibility of the imagination model might suggest that it is unfalsifiable, or lacks predictive power. However, a few things would show the model to be false. If it turned out that biases are much closer to the functional profile of some other state, say belief (sensitive to logical form, evidence responsive), that would suggest that a belief model would offer a better account than the imagination model. If there were good arguments against the possibility of unconscious imaginings, or against them having the functional role I claim for them, the model would be falsified insofar as unconscious imagination would be an inappropriate candidate on which to model bias. As for predictive power, it is here that details will matter: different kinds of imaginings may be predictive of different behaviour, and the more we learn about the operations of different kinds of unconscious imaginings, the more predictions we will be able to make about implicit biases understood in such terms.

19 Holroyd and Sweetman (2016) have argued that in giving accounts of the nature of implicit bias we risk working at too general a level, thus failing to attend to the heterogeneity present in this psychological category. They suggest that implicit biases may well differ both in terms of content and in terms of the processes underpinning them. Their discussion takes place in an associative framework—they argue against distinguishing associations according to whether they are semantic or affective (pp. 92–96), but suggest that the way different associations operate may be explained by differences in content and underpinning processes (p. 88). The way imagination can be involved in associative processing (where the components are imagistic or propositional) may be one way in which different underpinning processes are involved in associative processing. My model is wider than Holroyd and Sweetman call for though, insofar as it is also able to accommodate implicit biases which operate non‐associatively.

20 None of this is to say that gut feelings are always downstream of implicit biases. Just as we do not expect any given behaviour (e.g., seating distance from a member of the targeted group) to be associated with *every single instance* of implicit bias, neither should we expect gut feelings to be so.

21 See Jacob Berger (in press) for a similar view of how we are aware of our implicit attitudes.

22 I use the term *behaviour* rather than *action* to remain neutral on whether the downstream effects of implicit biases “rise to the level of actions”, rather than being effects which unfold “automatically and […] without awareness” (see Van Leeuwen, 2016, for discussion).

23 Recently, there has been some dissent. For example, Oswald and colleagues (2013), have claimed on the basis of their meta‐analysis of IAT results, that it is a “poor” predictor of subsequent behaviour. I note a handful of things in response (see IAT roundtable on the Brains Blog, and Brownstein, Madva, & Gawronski, manuscript, for discussion). First, in comparing Oswald and colleagues' meta‐analysis to their own earlier one (2009), Greenwald and colleagues argue that the differences between the two are due to a difference in method. Second, we ought not expect implicit measures to predict behaviour in all contexts (Friese, Hofmann, & Schmitt, 2008), but as Brownstein and colleagues (manuscript) point out, Oswald and colleagues' meta‐analysis was blind to relevant contextual factors in this respect (p. 5). Third, “both meta‐analyses aggregate correlational effect sizes that are large enough to justify concluding that IAT measures predict societally important discrimination” (Greenwald, Banaji, & Nosek, 2015, p. 559). In particular, both agreed “in expecting more than 4% of variance in discrimination‐relevant criterion measures is predicted by Black‐White race IAT measures” (Greenwald et al., 2015, p. 560). Greenwald and colleagues claim that Oswald and colleagues' conclusion “did not take into account that small effect sizes affecting many people or affecting individual people repeatedly can have great societal significance” (Greenwald et al., 2015, p. 560, see also Levy, 2015, p. 803).

24 The idea that imagination can guide behaviour has been widely defended (e.g., Doggett & Egan, 2007; Nanay, 2013; Van Leeuwen, 2011; Velleman, 2000), though it is controversial how and under what conditions it does so (see, e.g., Van Leeuwen, 2011; 2014, for a survey, c.f., Langland‐Hassan, 2012). Most typically, this view is motivated by pretense actions (see fn. 25), though it has also been invoked in explanations of other cases (see, e.g., Gendler's (2007) imagination account of self‐deceptive actions; Currie's (2000) imagination accounts of delusional actions, Ichino's (manuscript) imagination account of superstitious action, and Velleman (2000) for many other everyday cases).

25 Some philosophers have argued that in pretense, imaginings do not motivate action directly, but do so via belief. For example, Shaun Nichols and Stephen Stich argue that that which one imagines generates conditional beliefs, and pretense occurs when one desires to behave in line with the truth of the antecedent of those conditional beliefs (Nichols & Stich, 2003, pp. 37–38). However this view of pretense which generates the dormancy of imaginings makes pretenders far too sophisticated (Ichino, 2015, p. 62) and gives a depressingly adult‐like view of play (Velleman, 2000, pp. 257–258). In addition, we are able to act without having conditional beliefs about how we might behave if something were the case (see Van Leeuwen, 2011, p. 59, and discussion in Ichino, 2015, p. 64). Note though that Nichols and Stich's explanation of pretense as imagination motivating via belief might be right in some cases, so too might Van Leeuwen's account according to which both beliefs and imaginings guide pretense (Van Leeuwen, 2009; 2016). For my model of bias to be viable it only needs to be that there are some cases in which, to borrow a phrase from Van Leeuwen (2011), *imagination is where the action is.* Then that is enough to say the actions guided by implicit biases could be ones in which imagination is doing the work without belief, even if this is not so in cases of pretense.

26 Kelly and Roedder (2008) argue that implicit biases are bad regardless of behaviour (pp. 527–528). Some people will have the intuition that there is something wrong with imagining certain things even if doing so is causally isolated from any action one engages in subsequent to the imaginative episode. For example, if you think that my imagining *hurting your child* is morally problematic, even if you know that having this imagining will not affect my action in any way, then you already think that imaginative states can be morally problematic, and so you already think that my account can result in the right judgement about implicit biases. For those who think that imaginings being *unconscious* takes them outside of the moral domain, I note that this is something which all models of implicit bias share. If we cannot be held responsible for our biases because they are unconscious, or if biases cannot be appropriately labelled as morally wrong because they are unconscious, any model of them which honours their being unconscious will face the same problem.
